# Imaging-based biomarkers in acute pancreatitis: the predictive value of adrenal contrast ratios for intensive care unit admission

**DOI:** 10.1007/s00261-025-04931-x

**Published:** 2025-04-10

**Authors:** Sevde Nur Emir, Hasan Kumru, Gülbanu Güner, Aylin Acar, Tolga Canbak

**Affiliations:** 1 University of Health Sciences, Umraniye Training and Research Hospital, Department of Radiology, Istanbul, Turkey; 2 University of Health Sciences, Umraniye Training and Research Hospital, Department of General Surgery, Istanbul, Turkey

**Keywords:** Acute pancreatitis, Imaging, Computed tomography, Adrenal gland, Contrast media, Intensive care units, Risk assessment

## Abstract

**Background:**

Early risk stratification is crucial in acute biliary pancreatitis (ABP) to optimize patient management and guide intensive care unit (ICU) admission decisions. Traditional biomarkers and scoring systems have limitations in early severity assessment. This study aimed to evaluate the predictive value of adrenal contrast ratios on contrast-enhanced CT (CECT) as imaging-based biomarkers for ICU admission and prolonged hospitalization in ABP patients.

**Methods:**

This retrospective study included 288 ABP patients who underwent CECT within 24 h of admission. Adrenal-to-inferior vena cava (IVC) and adrenal-to-spleen contrast ratios were measured from portal venous phase images. The predictive performance of these ratios for ICU admission was assessed using receiver operating characteristic (ROC) analysis, and their correlation with clinical outcomes was evaluated through regression analysis.

**Results:**

ICU-admitted patients had significantly higher adrenal contrast ratios compared to non-ICU patients (adrenal-to-IVC ratio: 1.15 vs. 0.99, *p* < 0.001; adrenal-to-spleen ratio: 0.97 vs. 0.75, *p* < 0.001). ROC analysis demonstrated strong predictive accuracy (AUC = 0.74 for adrenal-to-IVC, AUC = 0.81 for adrenal-to-spleen). Additionally, adrenal contrast ratios correlated significantly with prolonged hospital stay (*r* = 0.49–0.55, *p* < 0.001).

**Conclusion:**

Adrenal contrast ratios serve as promising imaging-based biomarkers for early ICU admission prediction and risk stratification in ABP patients. Their integration into clinical decision-making may enhance early management strategies. Further prospective validation is warranted.

**Graphical abstract:**

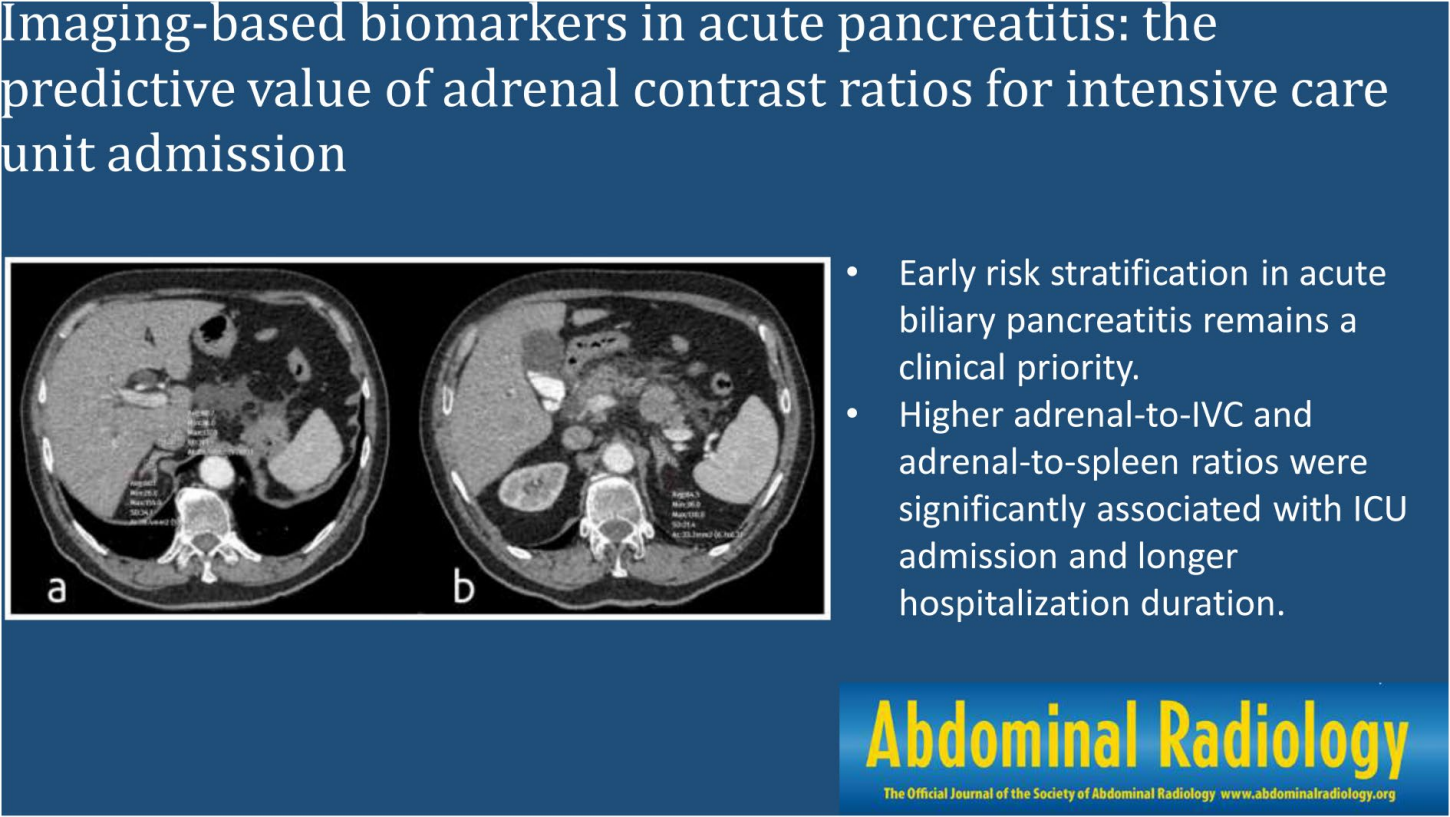

## Background

Acute pancreatitis (AP) is an inflammatory condition of the pancreas with a highly variable clinical course, ranging from mild self-limiting disease to severe multi-organ failure [1].

The 2012 Revised Atlanta Classification categorizes AP into interstitial edematous and necrotizing pancreatitis, with severity classified as mild, moderately severe, or severe based on the presence of organ failure and local/systemic complications. Early prediction of disease severity is crucial for optimizing patient management and guiding intensive care unit (ICU) admission decisions [2, 3].

Biliary pancreatitis, caused by gallstones, is among the most common etiologies of AP. In this subgroup, early risk stratification is particularly important to prevent complications and ensure timely Intensive care unit (ICU) interventions [4]. Several clinical and imaging-based scoring systems, such as the Ranson score, Acute Physiology and Chronic Health Evaluation II (APACHE-II), Bedside Index for Severity in Acute Pancreatitis (BISAP), and the Computed Tomography Severity Index (CTSI), have been developed to assess AP severity. However, these methods have significant limitations, including the need for late-stage imaging, complex calculations, or delayed laboratory results, reducing their applicability in early risk assessment [5–8].

Among these, the Balthazar CT Severity Index (CTSI) is one of the most commonly used imaging-based scoring systems, providing a structured approach for risk stratification by evaluating pancreatic inflammation and necrosis on contrast-enhanced CT (CECT) findings. However, Balthazar scoring has notable limitations, such as subjectivity in grading, lack of vascular parameters, and the requirement for late-stage imaging (48–72 h after onset) [7–9]. Moreover, Balthazar scoring does not incorporate systemic hemodynamic changes, which may play a crucial role in predicting disease severity and ICU admission. Given these challenges, there is a growing need for alternative imaging-based biomarkers that allow for earlier and more objective risk assessment, particularly for predicting ICU admission and prolonged hospitalization.

One of the major drawbacks of current scoring systems is delayed risk stratification, as many models require 24–48 h for full assessment, limiting their effectiveness in early triage [5, 6]. Additionally, complexity and late-stage imaging requirements pose significant challenges, particularly with CT-based scoring systems such as CTSI and Mortele CTSI, which require CECT at least 48–72 h after symptom onset, reducing their utility in the early phase of AP [7, 8]. Another important drawback is interobserver variability, as imaging-based scores rely on subjective interpretation, which may lead to discrepancies among radiologists [9]. Furthermore, these scoring systems do not assess real-time hemodynamic status, as they lack quantitative vascular imaging parameters that could provide a more accurate representation of systemic hemodynamic instability [10, 11].

Given these limitations, there is a growing interest in alternative biomarkers that offer rapid and reproducible assessment of disease severity. Various inflammatory markers such as C-reactive protein (CRP), neutrophil-to-lymphocyte ratio (NLR), platelet-to-lymphocyte ratio (PLR), mean platelet volume (MPV), and albumin levels have been explored as prognostic indicators in AP. Some studies suggest that incorporating CRP levels into existing scoring systems may enhance their predictive accuracy for severe AP. However, the predictive reliability of these markers remains inconsistent, necessitating the identification of more objective and reproducible early biomarkers [9–11].

Recent studies suggest that adrenal gland attenuation on contrast-enhanced CT may serve as a quantitative imaging biomarker for prognosis in critically ill patients. The adrenal-to-spleen ratio and adrenal-to-IVC ratio have been proposed as predictors of short and intermediate-term mortality in various acute conditions. These ratios provide a real-time assessment of adrenal perfusion relative to systemic circulation and have shown promise in identifying hemodynamic alterations associated with critical illness [12–14].

To date, no studies have systematically evaluated the role of adrenal contrast ratios in acute pancreatitis prognosis. This study aims to determine whether adrenal contrast ratios could predict ICU admission and prolonged hospitalization in patients with acute biliary pancreatitis. Identifying objective imaging-based biomarkers could improve early clinical decision-making and enhance risk stratification in AP management. This study also seeks to bridge the gap between current scoring systems and real-time hemodynamic assessment, providing a novel approach to early severity evaluation in AP.

## Methods

### Study design and patient selection

This retrospective study included 307 patients diagnosed with acute biliary pancreatitis (ABP) who were admitted to the general surgery department between January 2022 and October 2024. The diagnosis was based on clinical, laboratory, and imaging findings, and disease severity was classified according to the 2012 Revised Atlanta Classification:

- Mild AP: No organ failure, no local/systemic complications.
- Moderately Severe AP: Transient organ failure (< 48 h) and/or local complications.
- Severe AP: Persistent organ failure (> 48 h) involving respiratory, cardiovascular, or renal systems.

### Inclusion and exclusion criteria

Patients requiring hospitalization due to moderate/severe AP, persistent symptoms, or worsening inflammatory markers were included. ICU admission criteria encompassed persistent organ failure, hemodynamic instability, or severe respiratory compromise.

Patients without contrast-enhanced CT scans (*n* = 8) were excluded as adrenal gland, spleen, and IVC attenuation measurements require adequate contrast opacification for accurate evaluation. Additionally, patients with inappropriate contrast phases (*n* = 3) were excluded, as incorrect timing may lead to misleading attenuation values, potentially affecting the reliability of adrenal contrast ratio calculations. Patients with adrenal adenomas (*n* = 6) were also excluded, as these benign lesions may alter adrenal attenuation values and introduce variability in measurements. Furthermore, cases with significant motion artifacts (*n* = 2) were omitted to avoid inaccuracies in attenuation quantification. After applying these exclusion criteria, a total of 288 patients were included in the final analysis.

### Imaging acquisition and analysis

CECT was performed using a GE Optima 128-slice multidedector CT scanner. The imaging protocol included arterial and portal venous phases, acquired within 24 h of admission. The scanning parameters were as follows: 120 kVp, automatic tube current modulation, collimation 64 × 0.625 mm, and pitch 1.375. Intravenous contrast (iodine-based, 300 mg/mL) was administered at 4.0 mL/s through an antecubital vein, with bolus tracking set at 100 HU in the descending aorta. Portal venous phase images were obtained 65–70 s after contrast injection.

### Image analysis

Image analysis was conducted on a Picture Archiving and Communication System (PACS) Workstation utilizing region of interest (ROI) measurements to evaluate the attenuation values of the inferior vena cava (IVC), both adrenal glands, and the spleen during the portal venous phase on. Splenic attenuation was assessed at three axial levels using ROIs of 1–2.0 cm², while adrenal attenuation was measured at the confluence of both adrenal limbs with a preferred ROI size of 10–40 mm². The IVC attenuation was evaluated at the same axial level as the right adrenal gland. During splenic measurements, vascular structures and triangular hypodense areas suggestive of infarction at the splenic periphery were excluded from analysis.

The adrenal-to-IVC ratio was calculated as the mean adrenal gland attenuation (HU) divided by the attenuation of the IVC (HU). Similarly, the adrenal-to-spleen ratio was calculated as the mean adrenal gland attenuation (HU) divided by the mean spleen attenuation (HU). The formulas are as follows:

Adrenal-to-IVC ratio = Mean adrenal gland attenuation (HU) / IVC attenuation (HU).

Adrenal-to-Spleen ratio = Mean adrenal gland attenuation (HU) / Mean spleen attenuation (HU).

**Fig. 1:**
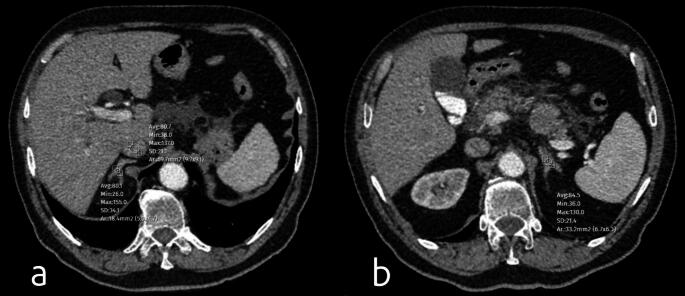
Axial contrast-enhanced CT images demonstrating attenuation measurements of the adrenal glands and inferior vena cava (IVC) in a 56-year-old male patient. The pancreas is enlarged and edematous, with increased density in the peripancreatic fat and localized fluid collections, consistent with Balthazar grade D pancreatitis. The patient did not require ICU admission. (**a**) Measurement of the right adrenal gland with an average attenuation value of 80.7 HU and the IVC with an average attenuation value of 80.1 HU. (**b**) Measurement of the left adrenal gland with an average attenuation value of 84.5 HU. Based on these measurements, the adrenal-to-IVC ratio was calculated as 1.03, while the adrenal-to-spleen ratio was 0.62

**Fig. 2:**
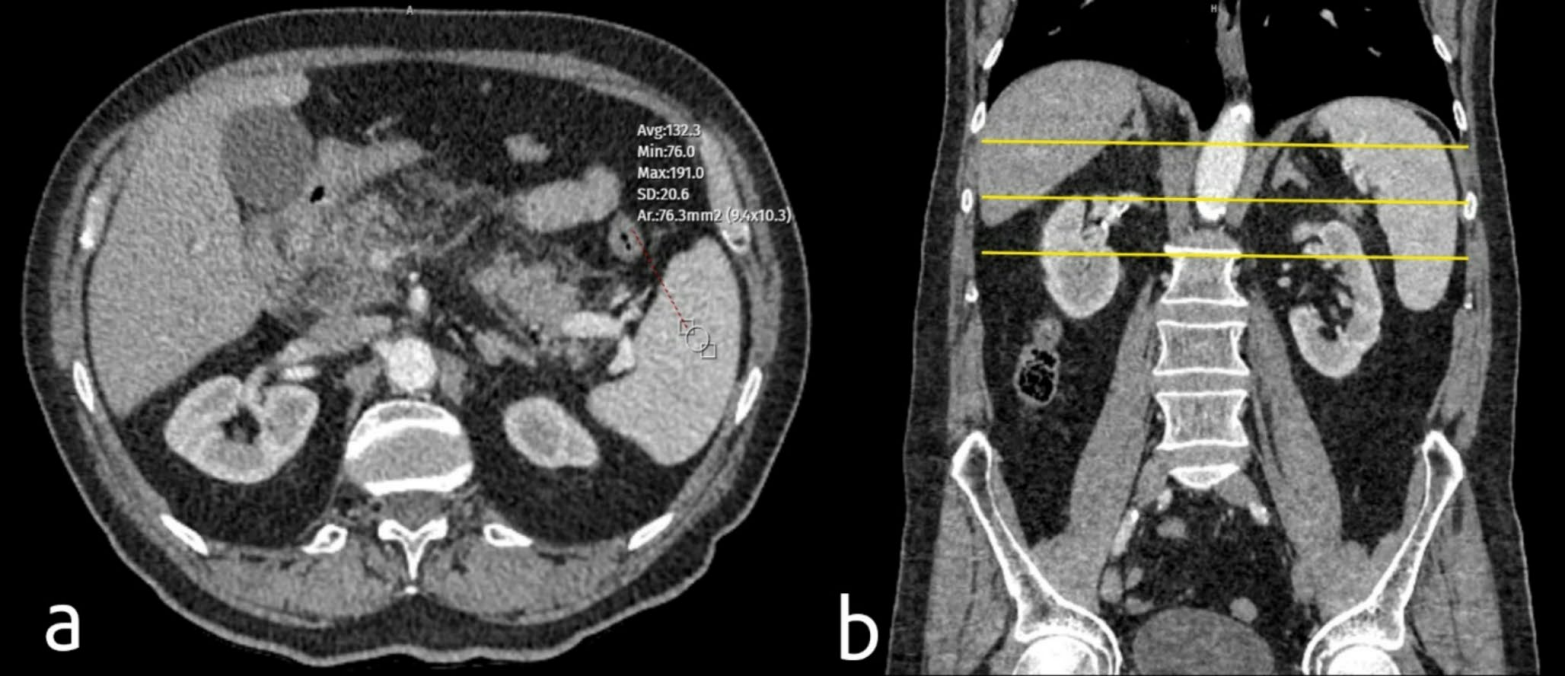
(**a**) Axial contrast-enhanced CT image showing a measurement of splenic attenuation with an average value of 132.3 HU. (**b**) Coronal contrast-enhanced CT image demonstrating the three axial levels (marked with yellow lines) where splenic attenuation measurements were performed. The mean splenic attenuation value was calculated using the average of these three measurements

Figures 1 and 2 illustrates how adrenal gland, IVC, and splenic contrast measurements are performed on portal venous phase contrast-enhanced CT, including splenic measurements obtained at three axial levels.

**Fig. 3:**
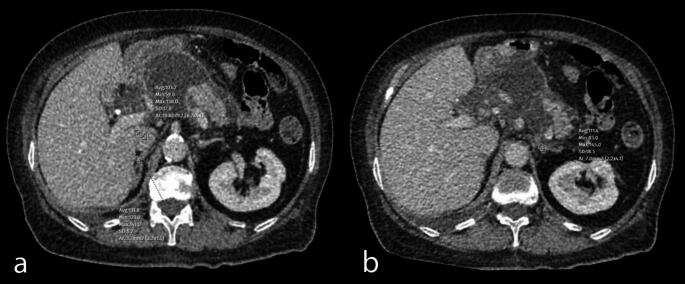
Axial contrast-enhanced CT images demonstrating attenuation measurements of the adrenal glands and inferior vena cava (IVC) in a 65-year-old female patient with acute biliary pancreatitis who required ICU admission.The pancreas was markedly enlarged and edematous, with extensive peripancreatic fat stranding and fluid collections. These findings correspond to Balthazar grade E, indicating severe disease with a high risk of complications. (**a**) Measurement of the right adrenal gland attenuation value of 131.8 HU and the IVC attenuation with value of 103.2 HU. (**b**) Measurement of the left adrenal gland with attenuation value of 111.4 HU. The mean spleen attenuation was measured as 101 HU but was not shown in the figure. Based on these measurements, the adrenal-to-IVC ratio was calculated as 1.18, while the adrenal-to-spleen ratio was 1.20. These values suggest an increased adrenal enhancement relative to the IVC and spleen, which aligns with findings in patients requiring intensive care unit admission

Figure 3 presents adrenal contrast measurements in a patient admitted to the ICU due to acute biliary pancreatitis, showing significantly elevated adrenal contrast ratios.

### Interobserver agreement

To assess the reproducibility of adrenal contrast ratio measurements, two radiologists with 11 and 9 years of experience, respectively, independently evaluated adrenal gland and reference organ attenuation values using a standardized ROI. Both radiologists performed the measurements blinded to patient prognosis and clinical outcomes.

Intraclass correlation coefficient (ICC) analysis was conducted to determine interobserver agreement and evaluate the reliability of the measurements.

### Balthazar score assessment

The Balthazar CT Severity Index (CTSI) was used to evaluate the severity of acute pancreatitis based on CECT findings. Each patient was assigned a Balthazar grade (A–E) based on pancreatic inflammation and necrosis, following the 2012 Revised Atlanta Classification criteria [7]. The scores were determined independently by two radiologists blinded to clinical outcomes, and in case of discrepancies, a consensus was reached.

### Statistical analysis

Statistical analyses were conducted using NCSS 2007 (Kaysville, Utah, USA). Descriptive statistics were reported as mean ± standard deviation (SD) for continuous variables and as percentages for categorical variables. Comparisons between ICU and non-ICU patients were performed using the Mann-Whitney U test for non-normally distributed continuous variables and the chi-square test for categorical variables. Correlations between adrenal contrast ratios, laboratory parameters, and clinical outcomes were assessed using Spearman’s correlation analysis. The predictive power of adrenal contrast ratios for ICU admission was evaluated using receiver operating characteristic (ROC) analysis, with area under the curve (AUC) calculations. Multivariate logistic regression was conducted to identify independent predictors of ICU admission, while linear regression analysis was used to examine the associations between predictive markers and hospital length of stay. A p-value of < 0.05 was considered statistically significant for all analyses.

This study was conducted in accordance with the Declaration of Helsinki and its subsequent revisions.

## Results

### Descriptive statistics

A total of 288 patients were included in the study, with an overall ICU admission rate of 20.1% (*n* = 58). The mean age of the study population was 59.27 years (SD: 17.13), with a gender distribution of 54.2% female and 45.8% male. The average hospital length of stay was 5.93 days (SD: 7.42, range: 0–56 days).

When comparing ICU-admitted and non-ICU patients, notable differences were observed in age and hospital length of stay, while gender distribution remained similar between groups. ICU-admitted patients were significantly older than non-ICU patients (67.0 vs. 58.7 years), suggesting that older age may be associated with an increased likelihood of requiring intensive care support. The proportion of female patients was nearly identical in both groups (55.6% in ICU vs. 55.0% in non-ICU patients), indicating that gender did not play a significant role in ICU admission.

As expected, ICU patients had significantly longer hospital stays compared to non-ICU patients (13.6 vs. 4.5 days, *p* < 0.001), reflecting the severity of their condition and the need for prolonged monitoring and treatment. Among ICU-admitted patients, the mean ICU length of stay was 7.8 days (SD: 6.2, range: 1–34 days), further underscoring the clinical burden associated with severe acute biliary pancreatitis.

Although the proportion of chronic disease was higher in ICU patients, statistical analysis using the chi-square test did not show a significant difference between ICU and non-ICU groups regarding chronic disease presence (*p* = 0.21).

Among the laboratory parameters, the mean CRP level was 98.5 mg/L (SD: 112.3), while the mean total bilirubin and lipase levels were 1.87 mg/dL (SD: 2.11) and 352.8 U/L (SD: 678.8), respectively (Table 1).

**Table 1:** Demographic and clinical characteristics of patients with acute biliary pancreatitis

Characteristics	Mean ± SD or %
Age (years)	59.27 ± 17.13
Gender (Female/Male)	54.2% / 45.8%
ICU Admission Rate	20.1% (*n* = 58)
Mean Hospital LOS (days)	5.93 ± 7.42
Mean CRP (mg/L)	98.5 ± 112.3
Mean Total Bilirubin (mg/dL)	1.87 ± 2.11
Mean Lipase (U/L)	352.8 ± 678.8

A total of 191 patients (66.3%) had at least one chronic disease, while 97 patients (33.7%) had no reported chronic conditions. The most frequently observed chronic comorbidities included hypertension (HT), diabetes mellitus (DM), coronary artery disease (CAD), chronic kidney disease (CKD), and chronic obstructive pulmonary disease (COPD). When comparing ICU-admitted and non-ICU patients, the proportion of patients with chronic diseases was higher in the ICU group (74.1% vs. 64.3%), although this difference did not reach statistical significance (*p* = 0.21).

While ICU-admitted patients had higher CRP levels compared to non-ICU patients (115.57 vs. 82.49 mg/L), this difference did not reach statistical significance (*p* = 0.08). Similarly, no significant differences were found in amylase, lipase, or bilirubin levels between ICU and non-ICU patients (*p* > 0.05 for all). A summary of the key laboratory parameter comparisons is presented in Table 2.

**Table 2:** Comparison of laboratory parameters between ICU and Non-ICU patients

Variable	ICU Patients	Non-ICU Patients	*p*-value
Mean CRP (mg/L)	115.57	82.49	0.08
Mean Total Bilirubin (mg/dL)	N/S	N/S	> 0.05
Mean Lipase (U/L)	N/S	N/S	> 0.05

### Imaging analysis & Balthazar score distribution

The mean right adrenal gland attenuation was 90.24 HU (SD: 21.13), while the left adrenal gland attenuation was 90.37 HU (SD: 20.45). The mean spleen attenuation was 117.46 HU (SD: 18.73), and the mean IVC attenuation was 134.17 HU (SD: 32.09).

The distribution of Balthazar scores among the study population showed that 9.27% of patients were classified as Grade A, while 21.62% were classified as Grade B. The most common category was Grade C, accounting for 35.91% of cases, followed by Grade D (17.37%) and Grade E (15.44%).

A significant correlation was observed between Balthazar scores and adrenal gland attenuation values. The correlation was statistically significant for right adrenal gland attenuation (*p* = 0.012) and left adrenal gland attenuation (*p* = 0.0009), while spleen attenuation did not show a significant association (*p* = 0.124).

Additionally, both adrenal-to-IVC and adrenal-to-spleen ratios showed significant correlations with Balthazar scores (*p* < 0.001 for both), suggesting that these adrenal contrast ratios could serve as biomarkers for assessing the severity of acute pancreatitis.

**Fig. 4:**
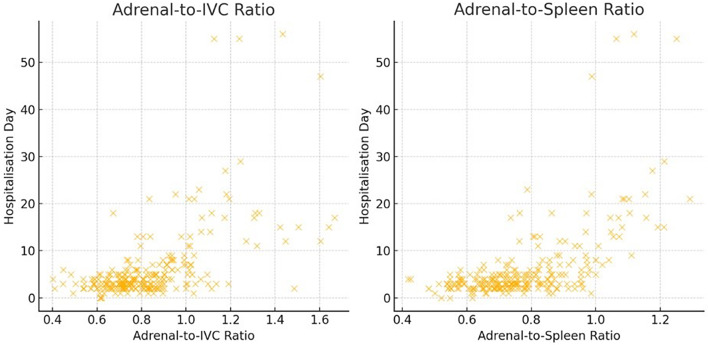
This boxplot illustrates the distribution of Adrenal-to-IVC ratios for each Balthazar score category (A, B, C, D, E). The ratios show a statistically significant association with the Balthazar scores (*p* < 0.001)

Figure 4 provides a boxplot illustrating the distribution of adrenal-to-IVC ratios across different Balthazar score categories, demonstrating a significant association with disease severity.

### Correlation between adrenal contrast ratios and clinical outcomes

A significant correlation was observed between adrenal contrast ratios and hospital length of stay. The adrenal-to-IVC ratio demonstrated a correlation coefficient of *r* = 0.49, *p* < 0.001, while the adrenal-to-spleen ratio showed an even stronger correlation (*r* = 0.55, *p* < 0.001).

ICU-admitted patients had significantly higher adrenal contrast ratios compared to non-ICU patients. The mean adrenal-to-IVC ratio was 1.03 ± 0.28 in ICU patients and 0.80 ± 0.19 in non-ICU patients (*p* < 0.001). Similarly, the adrenal-to-spleen ratio was significantly higher in ICU patients (0.93 ± 0.17 vs. 0.75 ± 0.15, *p* < 0.001).

### Predictive performance of adrenal contrast ratios

ROC analysis demonstrated that both adrenal-to-IVC and adrenal-to-spleen ratios were strong predictors of ICU admission. The adrenal-to-IVC ratio had an AUC of 0.74, with an optimal cut-off value of 1.01. Similarly, the adrenal-to-spleen ratio showed an AUC of 0.81, with a cut-off value of 0.86. These findings are summarized in Table 3.

**Table 3:** ROC analysis results for Adrenal-to-IVC and Adrenal-to-Spleen ratios in predicting ICU admission

Ratio	AUC	Optimal Cut-off Value	Sensitivity (%)	Specificity (%)
Adrenal-to-IVC Ratio	0.74	1.01	74.5	72.1
Adrenal-to-Spleen Ratio	0.81	0.86	81.3	78.4

Importantly, these findings suggest that patients with adrenal contrast ratio values above these cut-off points are at increased risk for ICU admission. This highlights the potential clinical applicability of adrenal contrast ratios as an early risk stratification tool for patients with acute biliary pancreatitis.

Additionally, individual adrenal gland attenuation values were analyzed for their predictive ability. The right adrenal gland attenuation demonstrated an AUC of 0.69 for ICU admission, with a significant correlation with hospital stay duration (*r* = 0.69, *p* < 0.001). Similarly, the left adrenal gland attenuation had an AUC of 0.65 and was also significantly correlated with hospital stay duration (*r* = 0.65, *p* < 0.001). In contrast, spleen attenuation exhibited poor predictive power for ICU admission (AUC = 0.43, *p* > 0.05) and showed no significant correlation with hospital stay duration.

**Fig. 5:**
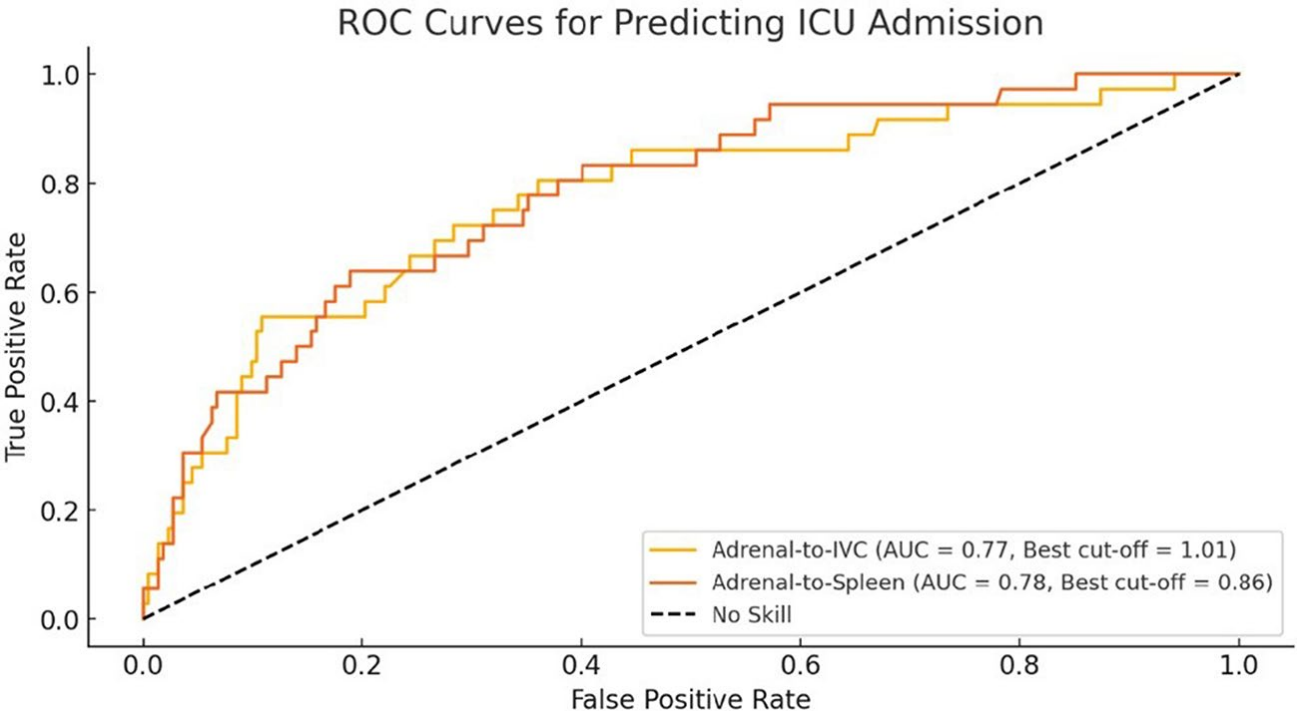
The ROC curves demonstrating the performance of the Adrenal-to-IVC and Adrenal-to-Spleen ratios in predicting ICU admission

Figure 5 displays the ROC curves illustrating the diagnostic performance of adrenal contrast ratios in predicting ICU admission.

**Fig. 6:**
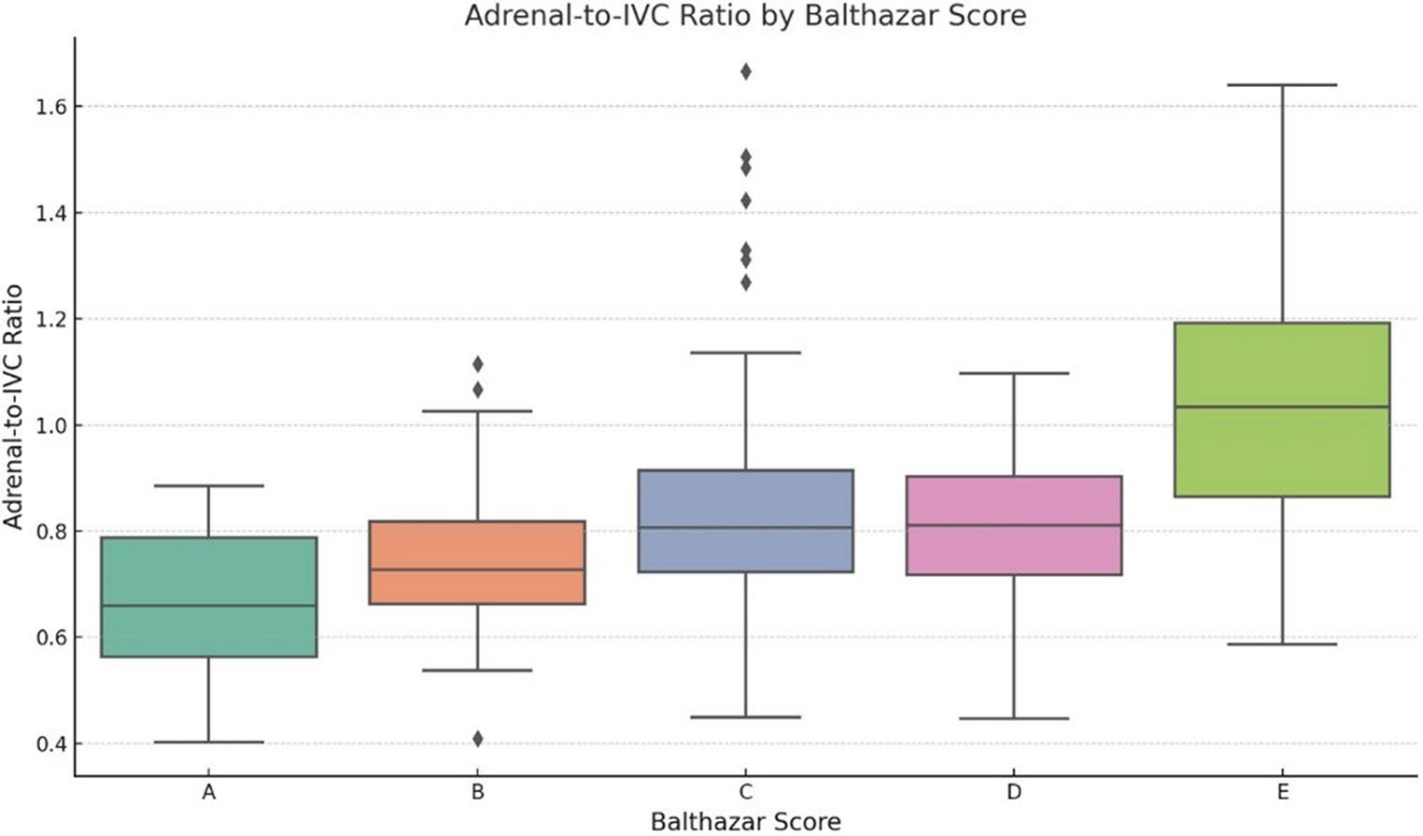
Scatter plots showing the relationship between Adrenal-to-IVC and Adrenal-to-Spleen ratios and hospital length of stay

Figure 6 presents scatter plots showing the correlation between adrenal-to-IVC and adrenal-to-spleen ratios and hospital length of stay.

### Multivariate analysis results

Multivariate logistic regression analysis was conducted to evaluate the independent predictive value of adrenal contrast ratios while adjusting for clinical and laboratory parameters.

The analysis identified age as a significant predictor of ICU admission (OR = 1.97, *p* = 0.018), indicating that increasing age is associated with a higher likelihood of ICU requirement. Although total bilirubin (OR = 2.21, *p* = 0.256) and direct bilirubin (OR = 0.36, *p* = 0.226) exhibited trends toward significance, their associations did not reach statistical significance.

Importantly, adrenal contrast ratios remained independent predictors of ICU admission, reinforcing their prognostic utility in early risk stratification of acute biliary pancreatitis patients.

**Fig. 7:**
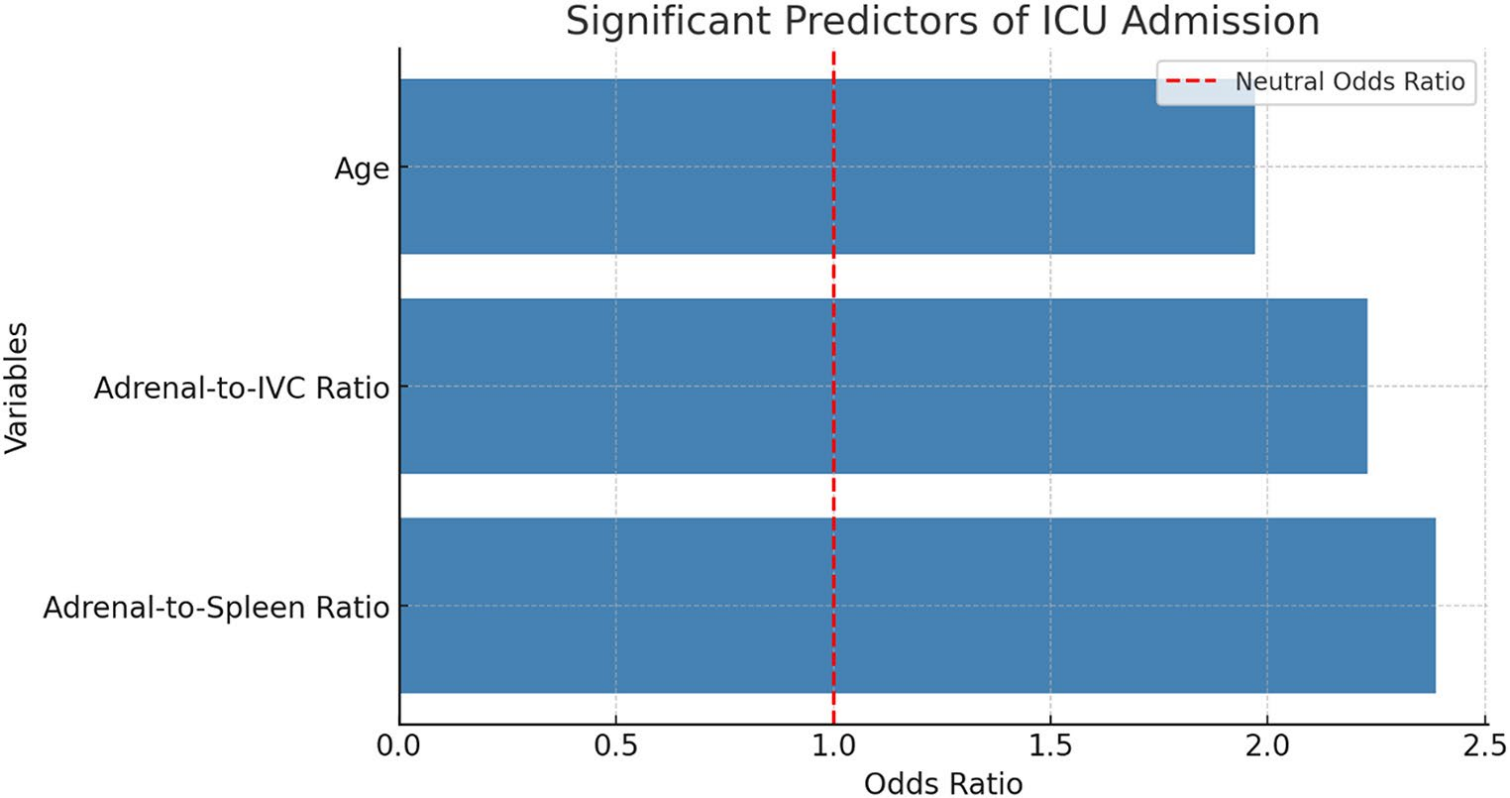
Multivariate logistic regression analysis results showing the significant predictors of ICU admission. Variables with a statistically significant impact (*p* < 0.05) are displayed

Figure 7 illustrates the significant predictors of ICU admission based on multivariate logistic regression analysis. Among the variables analyzed, age emerged as a statistically significant factor (OR = 1.97, *p* = 0.018), while adrenal contrast ratios remained independent predictors, reinforcing their prognostic value in acute pancreatitis patients.

### Interobserver agreement analysis

Intraclass correlation coefficient (ICC) analysis demonstrated strong inter-radiologist agreement in the measurement of adrenal gland and reference organ attenuation values. The right adrenal gland attenuation had an ICC of 0.85, while the left adrenal gland attenuation showed an even higher agreement with an ICC of 0.92. Similarly, the spleen attenuation exhibited an ICC of 0.88, and the IVC attenuation had an ICC of 0.84. For the calculated ratios, the adrenal-to-IVC ratio demonstrated an ICC of 0.81, and the adrenal-to-spleen ratio showed a high agreement with an ICC of 0.87.

These results indicate good to excellent inter-rater reliability, supporting the reproducibility of adrenal contrast ratio measurements.

## Discussion

Acute biliary pancreatitis (ABP) remains a clinically challenging entity due to its variable severity and potential for life-threatening complications. Identifying reliable prognostic markers is crucial for optimizing early management and resource allocation. Traditional laboratory markers, such as C-reactive protein (CRP), bilirubin, and lipase, have been widely used for disease severity assessment, yet their ability to predict ICU admission and prolonged hospitalization remains inconsistent [15, 16].

Our findings suggest that adrenal contrast ratios, particularly the adrenal-to-IVC and adrenal-to-spleen ratios, are independent predictors of disease severity in ABP. These quantitative imaging biomarkers may offer real-time, objective risk stratification, complementing conventional laboratory markers.

The IVC serves as a stable intravascular reference for contrast attenuation, reflecting circulating blood contrast density at the time of imaging. Prior studies have demonstrated that adrenal hyperattenuation relative to the IVC is associated with increased catecholamine release, a physiological response to severe stress states, such as shock. Conversely, the spleen exhibits hypoperfusion during systemic shock states, making the adrenal-to-spleen ratio an effective marker for hemodynamic instability. The combination of adrenal hyperattenuation and splenic hypoperfusion provides a broader assessment of circulatory stress, offering valuable prognostic insight into ICU admission risk [17].

Our study supports this pathophysiological link, as higher adrenal contrast ratios correlated with prolonged hospitalization, reinforcing the hypothesis that adrenal hyperattenuation reflects systemic stress responses in ABP patients. These findings align with previous research on critically ill populations, highlighting the potential of adrenal contrast parameters in severity assessment.

Our analysis revealed that older patients were significantly more likely to require ICU admission, suggesting that physiological reserve plays a role in critical illness progression. Although the proportion of patients with chronic diseases was higher in the ICU group, statistical analysis did not identify chronic disease as an independent predictor of ICU admission (*p* = 0.21). This suggests that while comorbidities contribute to overall prognosis, adrenal contrast ratios may provide a more direct assessment of disease severity in ABP.

Several studies have investigated the prognostic significance of adrenal gland hyperattenuation and adrenal contrast ratios in critically ill patients, yet their application in acute biliary pancreatitis (ABP) remains largely unexplored [12–14, 18, 19]. The findings of our study contribute to this growing body of literature by demonstrating the utility of adrenal contrast ratios as predictors of ICU admission and prolonged hospitalization in ABP patients. Our results are both consistent with and distinct from previous studies, emphasizing the need for disease-specific validation of adrenal contrast parameters.

Schek et al. [12] described adrenal hyperattenuation as part of the CT hypoperfusion complex, frequently observed in shock states and systemic hemodynamic instability. Our findings support a strong correlation between adrenal contrast ratios and prolonged hospitalization, suggesting that adrenal perfusion abnormalities may reflect systemic inflammatory stress in ABP. While previous studies examined adrenal hyperattenuation in trauma and general ICU cohorts, our study provides novel evidence of its prognostic value in pancreatitis-specific critical illness.

Boos et al. [13] demonstrated that qualitative hyperattenuation of adrenal glands on contrast-enhanced CT was associated with a higher mortality rate (50% vs. 16%) in ICU patients. Our findings align this, as adrenal contrast ratios were significantly higher in ICU-admitted ABP patients. However, while Boos et al. relied on visual assessment, our study used quantitative adrenal-to-IVC and adrenal-to-spleen ratios, improving objectivity and reproducibility. This reinforces the utility of adrenal contrast ratios as early severity markers in pancreatitis.

Winzer et al. [14]. identified an adrenal-to-spleen ratio cut-off of 1.37 as a predictor of 72-hour mortality in ICU patients. Our study found significantly lower cut-off values (0.86 for adrenal-to-spleen ratio and 1.01 for adrenal-to-IVC ratio), which likely reflects differences in patient populations. While Winzer et al. studied general ICU patients with diverse critical illnesses, our study focused specifically on ABP patients at an earlier stage of disease progression. This highlights the importance of disease-specific validation when applying adrenal contrast ratios to risk stratification models.

Pfister et al. [19] investigated the adrenal-to-spleen ratio in acute mesenteric ischemia (AMI), finding an association with 24-hour and 30-day mortality. However, their study identifie cut-off value (> 1.10 for 24-hour mortality) compared to our findings. This likely reflects pathophysiological differences—AMI is characterized by acute circulatory collapse, whereas ABP primarily involves inflammatory stress responses. Despite these differences, both studies underscore the prognostic value of adrenal contrast ratios in critically ill patients.

Our results suggest that patients with an adrenal-to-IVC ratio > 1.01 or an adrenal-to-spleen ratio > 0.86 should be considered at high risk for ICU admission. These individuals may benefit from closer hemodynamic monitoring, early ICU consultation, and aggressive supportive treatment strategies. Unlike traditional biomarkers such as CRP, which may exhibit delayed or inconsistent prognostic value, adrenal contrast ratios provide a real-time physiological assessment, making them a valuable addition to risk stratification models such as APACHE-II and BISAP.

Based on these findings, adrenal contrast ratios could be incorporated into early decision-making algorithms for ABP patients. Given their high predictive performance, particularly in ROC analysis, these ratios may help triage high-risk patients for closer monitoring or early ICU admission. Future prospective studies are needed to validate these findings and establish standardized cut-off values for clinical implementation.

Furthermore, our study demonstrated a significant correlation between adrenal gland attenuation values and Balthazar scores, aligning with previous literature on imaging biomarkers in acute pancreatitis. The integration of adrenal contrast ratios into established severity scoring systems may enhance their predictive accuracy for critical care decision-making.

Our study is the first to evaluate adrenal contrast ratios as imaging-based biomarkers for early risk stratification in ABP, highlighting their potential utility in identifying high-risk patients requiring ICU admission. Unlike conventional CT severity indices (e.g., Balthazar CTSI), which primarily assess local pancreatic changes, adrenal contrast ratios provide a broader assessment of systemic hemodynamic alterations, particularly in critically ill patients.

However, certain limitations should be acknowledged. First, this study was conducted at a single center with a retrospective design, which may limit the generalizability of the findings. Second, the sample size was relatively small, which could affect the statistical power of the analysis. Lastly, due to the low mortality rate in our cohort, mortality was not analyzed as an endpoint, unlike previous studies that investigated adrenal hyperattenuation in critically ill patients.

## Conclusion

Our study demonstrates that adrenal-to-spleen and adrenal-to-IVC contrast ratios are associated with ICU admission and prolonged hospital stay in ABP patients. These findings align with prior research on adrenal hyperattenuation as a prognostic marker in critical illness. Given their potential role in early risk stratification, adrenal contrast parameters warrant further prospective validation and possible integration into clinical decision-making algorithms.

## Data Availability

No datasets were generated or analysed during the current study.

## Abbreviations

ABP: Acute Biliary Pancreatitis
AUC: Area Under the Curve
AP: Acute Pancreatitis
APACHE-II: Acute Physiology and Chronic Health Evaluation II
BISAP: Bedside Index for Severity in Acute Pancreatitis
CECT: Contrast–Enhanced Computed Tomography
CKD: Chronic Kidney Disease
COPD: Chronic Obstructive Pulmonary Disease
CRP: C–Reactive Protein
CT: Computed Tomography
CTSI: CT Severity Index
DM: Diabetes Mellitus
HU: Hounsfield Unit
ICU: Intensive Care Unit
ICC: Intraclass Correlation Coefficient
IVC: Inferior Vena Cava
LOS: Length of Stay
MPV: Mean Platelet Volume
NLR: Neutrophil–to–Lymphocyte Ratio
OR: Odds Ratio
PLR: Platelet–to–Lymphocyte Ratio
PACS: Picture Archiving and Communication System
ROI: Region of Interest
ROC: Receiver Operating Characteristic
